# Characterization of speed adaptation while walking on an omnidirectional treadmill

**DOI:** 10.1186/s12984-020-00787-y

**Published:** 2020-11-23

**Authors:** Smit Soni, Anouk Lamontagne

**Affiliations:** 1 Virtual Reality and Mobility Laboratory, Jewish Rehabilitation Hospital site of CRIR–CISSS de Laval, 3205 Place Alton-Goldbloom, Laval, H7V 1R2 QC Canada; 2 School of Physical and Occupational Therapy, McGill University, 3654 prom Sir-William-Osler, Montreal, H3G 1Y5 Canada

**Keywords:** Gait, Locomotion, Kinematics, Electromyography, Virtual reality

## Abstract

**Background:**

Conventional treadmills are widely used for gait retraining in rehabilitation setting. Their usefulness for training more complex locomotor tasks, however, remains limited given that they do not allow changing the speed nor the direction of walking which are essential walking adaptations for efficient and safe community ambulation. These drawbacks can be addressed by using a self-pace omnidirectional treadmill, as those recently developed by the gaming industry, which allows speed changes and locomotor movements in any direction. The extent to which these treadmills yield a walking pattern that is similar to overground walking, however, is yet to be determined.

**Methods:**

The objective of this study was to compare spatiotemporal parameters, body kinematics and lower limb muscle activation of healthy young individuals walking at different speeds (slow, comfortable, fast) on a low-cost non-motorized omnidirectional treadmill with and without virtual reality (VR) vs. overground.

**Results:**

Results obtained from 12 young healthy individuals (18–29 years) showed that participants achieved slower speed on the treadmill compared to overground. On the treadmill, faster walking speeds were achieved by a mere increase in cadence, as opposed to a combined increase in cadence and step length when walking overground. At matched speed, enhanced stance phase knee flexion, reduced late stance ankle plantarflexion, as well as enhanced activation amplitudes of hip extensors in late stance and hip extensors in early swing were observed. The addition of VR to treadmill walking had little or no effect of walking outcomes. Collectively, results show that the omnidirectional treadmill yields a different walking pattern and lead to different adaptations to speed compared to overground walking. We suggest that these alterations are mainly driven by the reduced shear forces between the weight bearing foot and supporting surface and a perceived threat to balance on the omnidirectional treadmill.

**Conclusion:**

Since such treadmills are likely to be used for prolonged periods of time by gamers or patients undergoing physical rehabilitation, further research should aim at determining the impact of repeated exposure on gait biomechanics and lower limb musculoskeletal integrity.

## Background

Walking is essential to the completion of many activities of daily living [32] and has been identified as an important determinant of participation [8], quality of life [26] and health [12]. For these reasons, there is abundant research that describes the characteristics of walking, primarily level walking along a straight line, in both healthy and pathological populations across different age groups.

One of the major requirements of community mobility is speed adaptation [32]. Gait speed, considered as a “sixth vital sign”, directly correlates with functional ability [34] and balance confidence [20]. It has the potential to predict future health status [43], functional decline and mortality [14]. Walking speed reflects both functional and physiological changes and is a discriminating factor in determining potential for rehabilitation [13]. It further aids in prediction of falls and fear of falling [27]. Furthermore, progression of walking speed has been linked to clinical meaningful changes in quality of life as well as in home and community walking behavior [39].

Besides the obvious constraints on walking speed imposed by traffic lights, there are other subtle pressures to adjust speed while walking in the community, such as slowing down or speeding up to avoid an approaching obstacle [16]. Thus, one needs the ability to both increase and reduce walking speed to safely move around within the community. For these reasons, this study focused on the ability to walk at different speeds, including not only comfortable walking speed but also walking faster and slower walking speeds.

Unfortunately, gait-related mobility can be compromised by older age as well as by the presence of a neurological event such as stroke. A commonly used approach for gait rehabilitation is treadmill training, which allows the repeated practice of stereotyped, cyclical leg movements. This technology occupies a relatively small space and provides reliable speed control, sometimes with integrated body weight support system that facilitates stepping. Gait rehabilitation gains achieved through treadmill training, however, do not completely transfer to overground gait [4]. Furthermore, conventional treadmill training does not allow training important gait adaptations for community ambulation such as speed changes.

In the last decade, virtual reality (VR)-based training approaches for gait rehabilitation were proposed as a mean to allow patients training in meaningful and ecological environments that mimics the demands of everyday life, with the premise that such an approach would lead to better gait adaptation strategies and a better transfer of gains to everyday life [9]. Nowadays, VR systems for gait training usually consist of a visual display (e.g. rear-projection screen [35], helmet mounted display [24] or 3D monitor/screen [37]) coupled with a treadmill that allows the participant to train in real life scenarios. This technology remains limited, however, by the fact that it does not allow one to change direction while walking. For this reason, researchers as well as the gaming industry are currently working on the development of omnidirectional treadmills that allow changes in direction while accommodating gait speed changes. Coupled with the VR technology, omnidirectional treadmills provide visual motion information (optic flow) as experienced during overground locomotion, something that is not possible without VR given that the participant is stepping on the spot. This optic flow which provides information about the direction and speed generated by the relative motion between a participant’s eye and the immediate surroundings [31] plays an important role in adjusting one’s walking speed [25, 36] and walking trajectory [38, 46]. More research is needed, however, to determine the extent to which the gait pattern elicited on omnidirectional treadmills resembles that observed during overground locomotion. The latter consideration is important to ensure an optimal transfer of training gains to situations of everyday life, and to avoid unwanted gait movements that would ultimately lead to pain and injury.

To our knowledge, and given that omnidirectional treadmills are fairly recent, only one study carried out by [33] examined the influence of an omnidirectional treadmill on the walking pattern. The study, which used a motorized treadmill without VR, showed that torso and pelvis movements were similar on the treadmill vs. overground when turning while walking. However, the extent to which such findings can be extended to recent and low cost, non-motorized omnidirectional treadmills developed by the gaming industry remains unknown. Furthermore, how speed adaptations are achieved on omnidirectional treadmills and potential modulatory effects provided by optic flow through the virtual environment remain to be elucidated. In this research study, we specifically tested speed adaptions under three walking conditions, including walking on an omnidirectional treadmill with and without VR and walking overground. The two treadmill conditions were included to appraise the impact of the omnidirectional treadmill itself and the additional impact of VR which adds optic flow, the latter being shown to impact on temporal-distance factors and kinematics of gait [41].

Specifically, the objective of this study was to compare spatiotemporal parameters, body kinematics and lower limb muscle activation patterns while walking at different speeds on the omnidirectional treadmill with and without VR vs. overground. We hypothesized that as participants adapt their speed on the omnidirectional treadmill, they maintain a faster cadence and a shorter step length compared to when walking overground. It was further hypothesized that adding VR to omnidirectional treadmill would yield a walking pattern that more closely resembles that observed during overground gait compared to when walking on the omnidirectional treadmill without VR.

## Methods

### Participants

A convenience sample of 12 healthy young adults between the ages of 18 and 29 years participated in the study (5 females and 7 males; participants’ age = 24.4 ± 2.3 years (mean ± 1SD); comfortable walking speed = 1.42 ± 0.16 m/s as per the 10 m Walk Test [5]). The demographic data of participants are reported in Table 1. All participants had normal or corrected-to-normal visual acuity, as measured by a score equal or above to 20/20 on the EDTRS visual acuity chart [19], and intact cognition, as per a score ≥ 26 out of 30 on the Montreal Cognitive Assessment [29]. Participants were excluded if they presented any condition interfering with locomotion (e.g. orthopedic, rheumatologic or neurological), lower limb or back pain, as well as any visual condition interfering with 3D or color vision (e.g. strabismus, color blindness, etc.) The experiment was approved by the Research Ethics Board of the Centre for Interdisciplinary Research in Rehabilitation of Greater Montreal (CRIR) and all participants gave their written informed consent prior to entering the study.

**Table 1:** Participant characteristics

Participant	Gender	Age (years)	Height (cm)	Weight (kg)	Hand dominance
1	Male	18	178	95	Right
2	Female	23	160	56	Right
3	Male	28	174	70	Right
4	Female	24	163	77	Right
5	Male	26	175	70	Right
6	Female	22	153	59	Right
7	Female	25	170	51	Right
8	Male	26	170	72	Right
9	Female	28	168	70	Right
10	Male	27	178	72	Right
11	Male	29	165	79	Right
12	Male	29	178	69	Right
Mean (± 1SD)	–	25. 4 (3.3)	169.3 (8.0)	70.1 (11.5)	**–**

### Instrumental set up and procedure

The experiment took place at the Virtual Reality and Mobility Laboratory of the Jewish Rehabilitation Hospital-CISSS-Laval in one session lasting approximately 3 h, including preparation time and data collection. In addition to the assessment of characteristics listed in the participant section, anthropometric measurements (weight, height as well as segments length and width) were collected and participants were questioned as to whether they had any previous exposure to omnidirectional treadmill which lasted more-than one hour, as well as the frequency at which they play videogames per week and the time spent interacting with virtual environments (VEs) in the past three months. Participants were then requested to walk at different speeds under three *locomotor conditions*, in a random order: (1) walking overground without VR; (2) walking on the omnidirectional treadmill with VR and (3) walking on the omnidirectional treadmill without VR. *Speed conditions*, also presented in a random order, included walking at comfortable (measured a priori using the 10 m walk test overground), slow (66% of comfortable speed) and fast speed (133% of comfortable speed). Three blocks of 4 trials (4 trials X 3 speed conditions) yielding a total of 12 trials per locomotor condition were collected, for a grand total of 36 trials (12 X 3 walking conditions). Participants were allowed to rest between trials, as necessary.

For all 3 locomotor conditions, full body kinematics was recorded using a 12-camera Vicon-512™ motion capture system (Vicon Motion Systems LTD, UK). Forty passive reflective markers were placed on specific body landmarks on the participant, as specified in the Plug-In-gait model from Vicon™ [18]. The position of the markers was recorded at a sampling rate of 120 Hz and stored for offline analyses. Additionally, muscle activation was recorded using an 8-channel electromyography (EMG) system (Noraxon, USA) and by placing adhesive surface electrodes (silver-silver chloride, 1cm^2^ area, 1 cm inter-electrode spacing) bilaterally on four muscle groups of interest: rectus femoris (RF), semitendinosus (ST), tibialis anterior (TA) and medial gastrocnemius (MG). The skin was shaved, as needed, and cleaned with alcohol prior to apposing the surface electrodes. The pre-amplified EMG data was collected at 1080 Hz in Vicon™. The paragraphs below describe the specifics of each locomotor condition.

#### Walking overground without VR

**Fig. 1:**
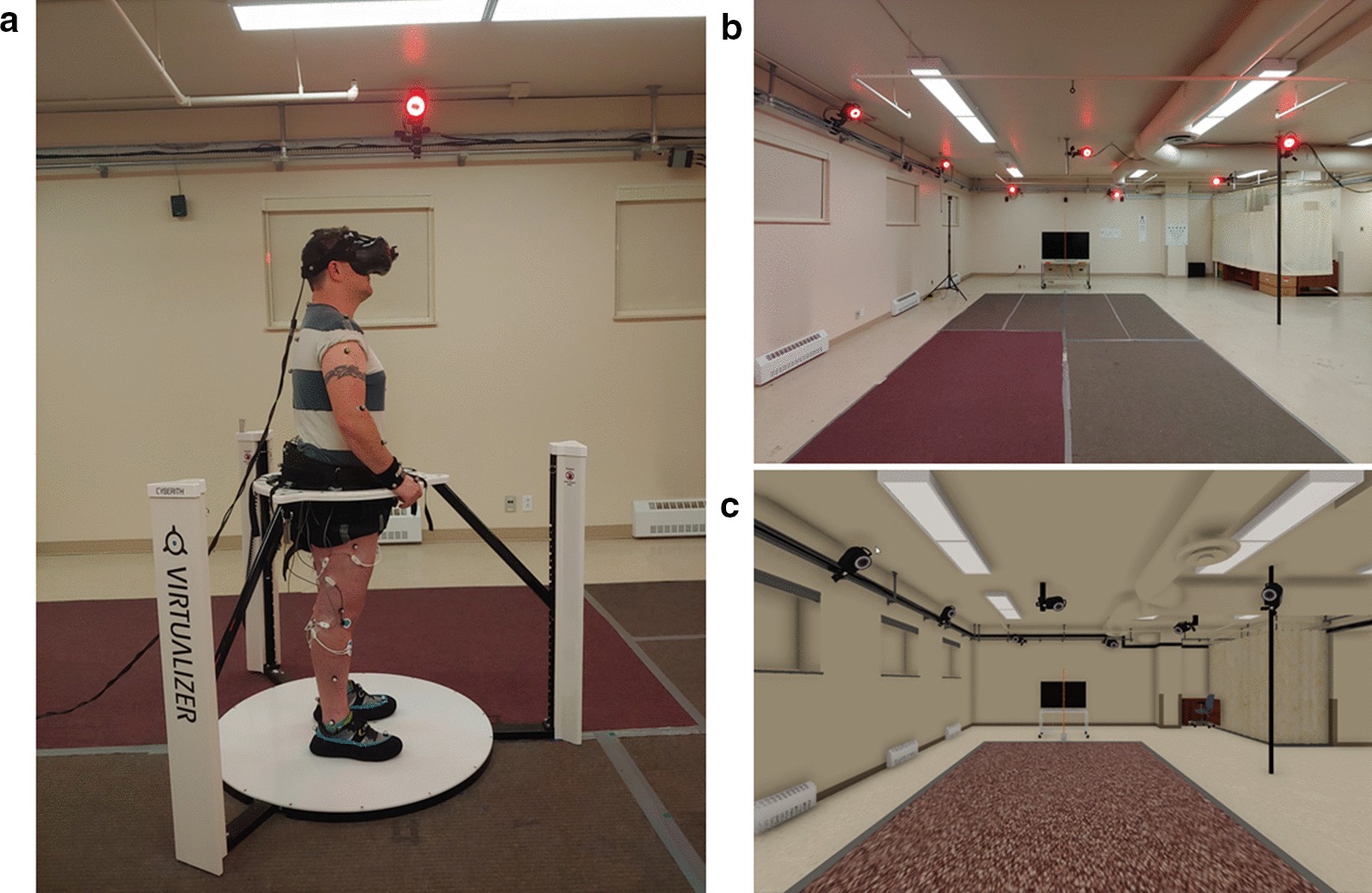
Omnidirectional treadmill with HTC Vive™ (**a**). Environment as viewed from the start position of the participant overground (**b**). Virtual environment as viewed in the HTC Vive™ (**c**)

The physical environment (PE) consisted of a 28.68 m^2^ area (7.8 m × 3.7 m) that was free of obstacles (Fig. 1). Participants were first provided practice until they felt comfortable walking at each of the targeted speeds. During this practice, a Vive™ controller (HTC, Taiwan) tethered to the participant’s pelvis was used to provide real-time feedback on the participant’s instantaneous walking speed. Once habituation was completed, participants completed the experimental trials. At the beginning of each trial, they were positioned at one end of the walking area, facing a television (TV) screen located straight ahead (0°) in the far space (8.5 m from start position and at a height of 1 m). The TV display was used to inform participants about the speed condition to be performed (slow, comfortable, and fast) and to deliver the start and stop cues of the walking trials. The stop cue was provided after 6 m of forward displacement, based on the position of the VIVE **™** controller.

#### Walking on the treadmill with VR

Participants were assessed while walking on the Virtualizer™ (Cyberith, Austria) omnidirectional treadmill and immersed in a VE that replicated the dimensions and features of the real-world laboratory and which also included the TV display. Participants wore a special low friction slippers over the shoes and a harness with no body weight support. The Virtualizer contains six optical sensors located in the center of a 100 cm diameter walking surface, which determines the walking displacement of participants. Sensors on a ring which goes around the torso track the orientation of participants in space. This information is fed in real time to the Unreal ™ gaming engine to update the participant position and orientation within the VE. The VE was viewed using the HTC VIVE™ (HTC, Taiwan) head-mounted display (HMD). This HMD weights 470 g and has a field of view of 110^o^ diagonal with resolution of 2160 × 1200 pixels and a refresh rate of 90 Hz. The position and orientation of the head tracked through the Vive’s HMD was also fed to Unreal 4™ (Epic games, USA) for a real-time update of the participants’ camera view within the VE. Together, the information provided by the treadmill and HMD allowed a decoupling of the participant’s direction of walking and head orientation within the VE.

Prior to collecting experimental trials, participants were provided habituation by walking on the omnidirectional treadmill with and without VR until they felt comfortable walking without holding the ring surrounding the treadmill.

#### Walking on the treadmill without VR

Participants walked on the omnidirectional treadmill without the HMD, while receiving the instructions and cues on the TV display, as described for the overground walking condition. They were provided a priori habituation as for the treadmill walking condition with VR condition, but without wearing the HMD.

### Data analysis

**Fig. 2:**
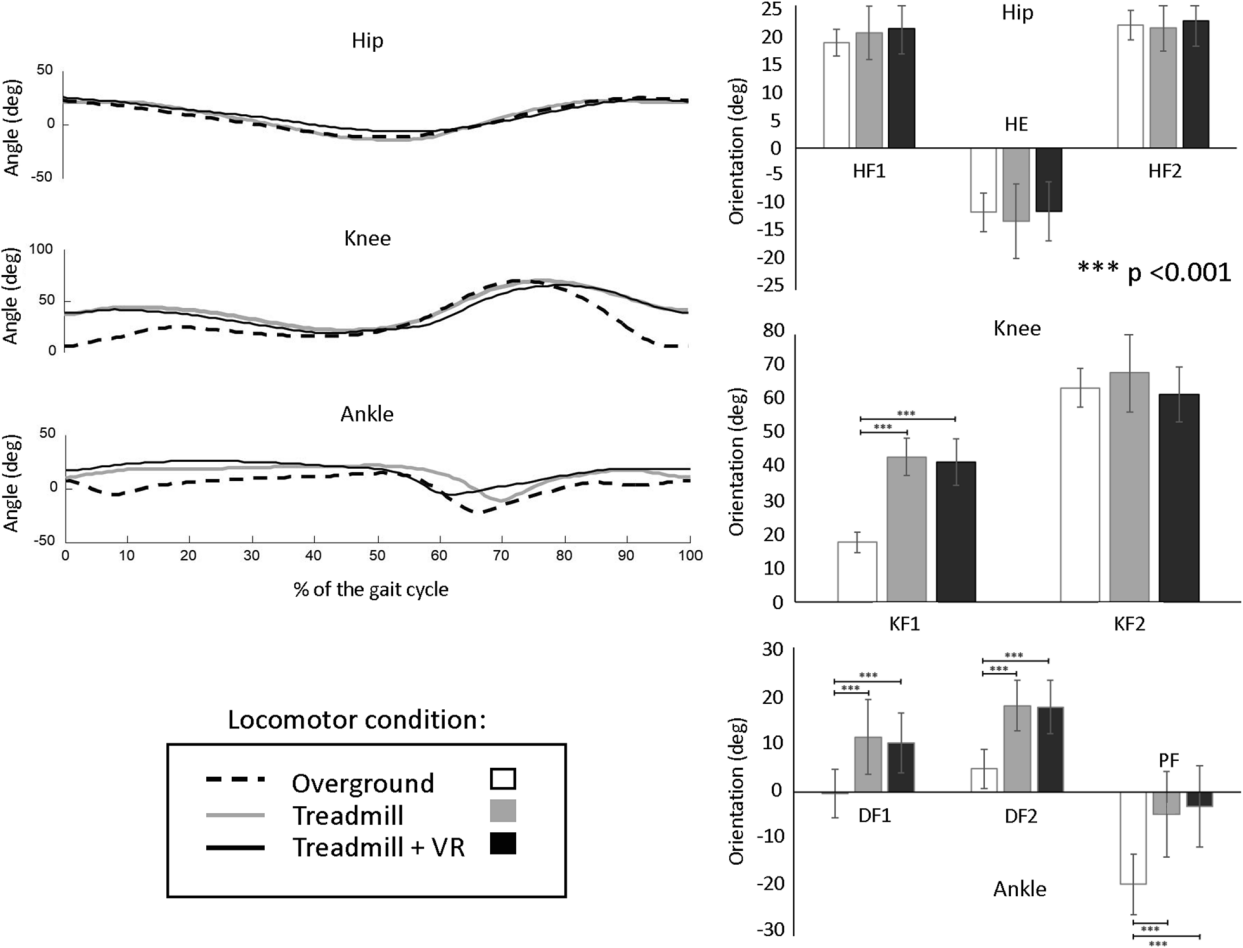
On the left: Hip, knee and ankle joint orientation in the sagittal plane as a function of gait cycle in one representative participant. Note the similarities in the hip profile, whereas the knee and ankle joint presented differences which are further depicted in the bar graphs on the right. On the right: Mean values (± 1SD) for the sagittal orientation of hip, knee and ankle at specific times of the gait cycle. Values for the hip are peak hip flexion in early stance (HF1; [between 0 and 30% of gait cycle]), peak hip extension (HE; [40–70%]) and peak hip flexion during swing (HF2; [70–100%]). For the knee, maximal knee flexion during the stance phase [KF1; 0–40%] and the swing phase [KF2; 60–100%] are illustrated. For the ankle, values are peak dorsiflexion at heel strike (DF1; [0%]), peak dorsiflexion during mid stance (DF2; [20–60%]) and peak plantarflexion during late stance (PF; [60–80%]). Statistically significant main effects are indicated, as applicable

Kinematic data was first labeled using Vicon Nexus software. The Vicon Plug-in-Gait model was used to compute segment orientations, displacements, and center of mass (CoM) displacement. All data, including the Plug-in-Gait output and the original marker locations was imported in Matlab R2016b for further analyses. Kinematic data were filtered using a dual-pass, 2nd order low-pass Butterworth filter with a cut off frequency of 10 Hz. The EMG data was band-pass filtered (10–400 Hz), rectified and smoothed at 20 Hz [23]. Lower limb (hip, knee and ankle) excursion of movement was calculated in all three planes. The orientation of lower limb joints were examined at specific times of the gait cycle, as illustrated in Fig. 2. For the hip joint, we included peak hip flexion during early stance (HF1; [between 0 and 30% of gait cycle]), peak hip extension (HE; [40–70%]), peak hip flexion during swing (HF2; [70–100%]). Maximal knee flexion was measured during the stance [KF1; 0–40%] and swing phases [KF2; 60–100%] of gait. For the ankle, we examined peak dorsiflexion at heel strike (DF1; [0%]), peak dorsiflexion during mid stance (DF2; [20–60%]) and peak plantarflexion during late stance (PF; [60–80%]). To calculate walking speed across the different walking conditions and since the body is not progressing forward during treadmill walking, the displacement heel and toe markers along the anteroposterior and mediolateral axis were used [21]. Step length was calculated as the displacement between successive contralateral heel contacts in the direction of progression. Cadence was obtained by computing the number of steps per minute. Swing time was calculated as the time interval between ipsilateral toe off and heel strike. Muscle activation amplitude was obtained using EMG integrals which were computed from linear envelopes for functionally-relevant time windows of the gait cycle, as described earlier [23]: MG activation at push-off [30%:70%], TA activation at toe-off [60%:80%], ST activation in early stance [0%:30%], and RF hip flexion burst at toe-off [60%:80%]. Each outcome was measured for every gait cycle before being averaged across gait cycles and trials of the same locomotor and speed condition for each participant.

### Statistical analysis

Generalized estimating equation model (GEE) were used to analyze all outcomes. The model was comprised of 2 within-subject factors accounting for locomotor condition (overground with no VR, treadmill with VR, treadmill with no VR) and walking speed (slow vs. comfortable vs. fast). GEEs were followed by Tukey post-hoc tests with Bonferroni adjustments in the case of significant main or interaction effects. Statistical analyses were performed in SAS® 9.4 software and the level of significance was set at ρ < 0.05.

## Results

### Participants

Most participants reported using videogames or simulators previously, that is either once a year (n = 6) or once a month (n = 4). Four had been previously exposed to virtual environments in the past three months but none had any previous exposure to walking on an omnidirectional treadmill.

### Speed adaptation task

**Fig. 3:**
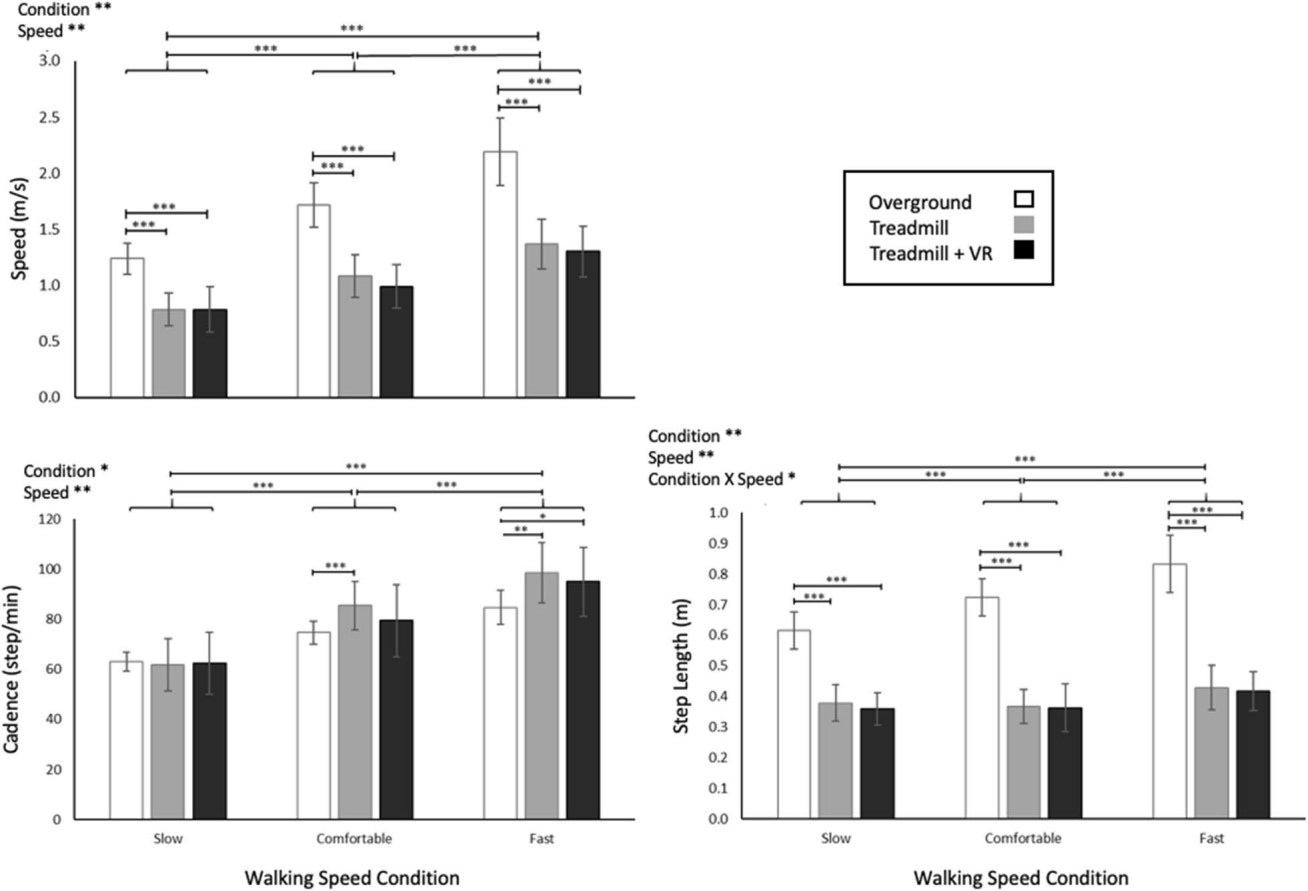
Average values (± 1SD) for gait speed, step length and cadence. Statistically significant main and interaction effects are indicated, as applicable. Likewise, post-hoc comparisons that were statistically significant are also illustrated. *p < 0.05. **p < 0.01. ***p < 0.0001

Figure 3 illustrates the average results for gait speed, step length and cadence. The GEE analysis revealed a significant main effect of locomotor condition for gait speed (X^2^ (2107) = 11.41, p = 0.0033) and cadence (X^2^ (2107) = 7.68, p = 0.021). Additionally, a significant main effect of walking speed condition was found on gait speed (X^2^ (2107) = 11.75, p = 0.0028) and cadence (X^2^ (2107), = 11.57, p = 0.0031). Post-hoc analyses showed that for a given speed condition, participants walked slower on the treadmill with or without VR compared to overground (range of difference = 0.64–0.69 m/s, p < 0.0001). A higher cadence was also observed on treadmill with VR in comparison to overground (p = 0.003) and treadmill without VR (p = 0.02). Neither gait speed nor cadence showed interaction effects. For step length, an interaction effect of locomotor condition and walking speed (X^2^ (4107), = 10.60, p = 0.031) was observed, with shorter step length on the treadmill with and without VR vs. overground (p < 0.0001). Note that no differences were observed for the treadmill conditions with vs. without VR in any of the outcomes mentioned above (p = 0.07 to 0.9).

Given the differences in walking speed across locomotor conditions and the well-recognized impact of walking speed on gait outcomes, all further comparisons reported below were completed at speed-matched conditions, that is while comparing outcomes measured during the ‘overground slow’ and ‘treadmill fast’ conditions which showed no significant differences (p = 0.06). This speed-matched analysis showed significantly higher cadence (p < 0.0001), shorter step length (p < 0.0001) as well as shorter swing time (p < 0.0001) but longer stance time (p < 0.0001) on the treadmill with and without VR (p < 0.0001) vs. overground. No differences were observed in the latter outcomes when comparing treadmill walking with vs. without VR (p = 0.17 to 0.68).

The comparison of joint angles at specific points during gait cycle is illustrated in Fig. 2. There were no statistically significant differences found for hip orientation or total hip excursion between locomotor conditions (p = 0.26 to 0.47). For the knee joint, a main effect of walking condition for KF1 (X^2^ (2,36), = 11.59, p = 0.003) and for total knee excursion was observed (X^2^ (2,36), = 9.01, p = 0.011). Post-hoc analyses showed that KF1 was larger for treadmill with and without VR compared to overground (p < 0.0001). Total knee excursion, however, was lower on the treadmill with (p < 0.0001) and without (p = 0.003) vs. overground. No significant difference was found for KF2 across locomotor conditions (p = 0.47). For the ankle joint, a main effect of locomotor condition was found for DF1 (X^2^ (2,36), = 9.79, p = 0.007), DF2 (X^2^ (2,36), = 11.06, p = 0.004) and PF (X^2^ (2,36), = 9.47, p = 0.008), but not for total ankle excursion (p = 0.07). Post-hoc analyses revealed significantly larger dorsiflexion angles (DF1 and DF2) and smaller plantarflexion angle (PF) on the treadmill with and without VR compared to overground (p < 0.0001).

**Fig. 4:**
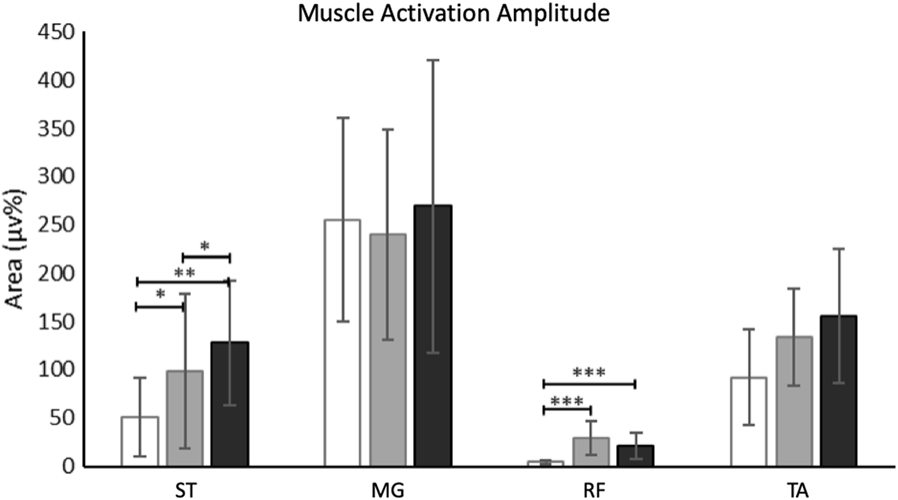
Average (± 1SD) muscle activation amplitudes recorded across locomotor conditions as participants are walking at matched speed. Activation amplitude were calculated for the semitendinosus (ST) in early stance [0–30%], medial gastrocnemius (MG) at push-off [30–70%], rectus femoris (RF) at hip flexion burst at toe off [60–80%], tibialis anterior (TA) at toe-off [60%—80%]. Statistically significant differences are indicated, as applicable. *p < 0.05. **p < 0.01. ***p < 0.0001

When comparing muscle activation amplitudes across locomotor conditions (Fig. 4), a main effect of locomotor condition was found for hip muscles, that is ST (hip extensor) activation in early stance (X^2^ (2,35), = 7.41, p = 0.025) and RF (hip flexor) activation at toe off (X^2^ (2,36), = 8.74, p = 0.01), but not for other muscle groups such as MG activation at push off and TA activation at toe off (p = 0.63- 0.06). Post-hoc analyses revealed that ST activation was larger during treadmill walking with VR (p = 0.001) compared to overground walking, and larger during treadmill walking with vs. without VR (p = 0.03). RF activation was larger during treadmill walking with without VR compared to overground (p < 0.0001), with no differences between treadmill conditions (p = 0.12).

## Discussion

Low cost omnidirectional treadmills with and without VR are becoming increasingly available and show promise for training clinical populations on complex locomotor tasks, as required for community ambulation such as modulating the speed or direction of walking in restricted space. Such combination of equipment allows for controlled, safe and repeated practice in ecological environments that are difficult to recreate in the laboratory or clinical setting. The omnidirectional feature of the treadmill also provides the option of changing the walking direction which is something that conventional treadmills do not allow and, if validated, could help train people with trajectory adaptation tasks.

Present findings show that ‘low-cost’ omnidirectional treadmills as the one tested in the present study impact on the biomechanics of gait, including temporal-distance parameters, lower limb kinematics and muscle activation. The addition of VR to treadmill walking induced limited differences, suggesting that the treadmill itself is the main contributing factor to alterations in gait biomechanics during VR-based omnidirectional treadmill walking.

Previous studies that investigated the effect of walking speed on the various temporal-distance factors of walking showed that faster walking speeds are achieved by decreasing the step duration (e.g. increasing cadence) and by increasing the step length. As speed increases, step length can only contribute up to a certain limit after which only cadence can be increased [28]. As further detailed in Table 2, our results indicate that while participants increased both step length and cadence when progressing from slow to comfortable to fast speed during overground walking, they increased their speed during treadmill walking mainly by increasing cadence and showed little to no changes in terms of step length. Furthermore, participants generally achieved slower speeds on the treadmill compared to overground, due to shorter step length and despite of a higher cadence (for comfortable and fast speed). Interestingly, once controlling for speed, those alterations in step length and cadence between the two walking conditions persisted.

**Table 2:** Temporal distance factors while walking overground vs. on the treadmill with and without VR

Outcome measure	Overground	Treadmill	Treadmill + VR
Speed (m/s)	1.24 ± 0.14	1.37 ± 0.22	1.30 ± 0.23
Cadence (steps/min)	63.16 ± 3.86	98.49 ± 12.02***	95.04 ± 13.74***
Step length (m)	0.62 ± 0.06	0.43 ± 0.07***	0.42 ± 0.06***
Stance time (%)	51.56 ± 6.38	65.72 ± 2.42***	66.00 ± 2.47***
Swing time (%)	49.78 ± 8.02	34.67 ± 2.54***	34.48 ± 2.75***

*** Level of significance p < 0.001

It should first be noted that the dimension of the treadmill, which was 100 cm in diameter, does not appear to explain the shorter step length during treadmill walking, given that the maximal step length that was observed during overground gait by the same participants was between 0.65 m and 0.8 m. Instead, we suggest that this shorter step length, as well as several other alterations in terms of temporal-distance factors, lower limb kinematics and muscle activation, are largely due to the low-friction walking surface of the treadmill and slippers which caused reduced shear forces between the weight bearing foot and supporting surface and lead to a perceived threat to balance. Indeed, the low friction between the foot and walking surface may have prevented participants from exerting a full ankle ‘push off’ in late stance, resulting in shorter step length. This reduction in step length was thus compensated, although not fully, by a faster cadence. In support of this hypothesis, participants in this study did show a significant reduction in late stance ankle plantarflexion on the treadmill vs. overground. Similar reductions in step and/or stride length [7, 10, 44] and in gait speed [44], as well as faster cadence [7], were also observed when walking on a slippery surface or while wearing footwear with lower friction insoles. People walking on slippery surfaces in simulated construction worksites, as participants of the present study walking on the omnidirectional treadmill, were also shown to adopt longer stance and shorter swing durations, as well as modified ankle joint kinematics, which altogether were suggested to represent gait adaptations that aim at preventing a slip [10].

Present findings also revealed that participants walking on the treadmill showed a more pronounced knee flexion in mid-stance, as well as both early- and mid-stance ankle dorsiflexion compared to overground gait. This kinematic pattern, which is typical of a crouched gait pattern [42], is consistent with the shorter step length displayed by the participants and may have served the purpose of maintaining the participants’ CoM at a lower position and hence maximize balance, as observed earlier during conventional treadmill walking [1].

The present study also revealed significantly higher amplitudes in muscle activation in most muscle groups during treadmill vs. overground walking. Such observation is consistent with previous reports of higher muscle activation amplitudes in the lower limbs while walking on the treadmill compared to overground [2]. The respectively larger activations in hip extensors (ST) and hip flexors (RF) in early stance and early swing may at first appear surprising, given the similar hip kinematic profiles observed between the locomotor conditions, as well as the smaller step length observed during the treadmill walking condition. Also, ankle plantarflexor activation at push-off (MG) did not differ between conditions but the corresponding peak plantarflexion amplitude was smaller during treadmill walking. These apparent discrepancies may be explained by a possible co-contraction between the flexor and extensor muscles around the hip and ankle joints. In the present case, and although habituation trials were provided prior to data collection, walking on an omnidirectional treadmill was new to all participants. Past studies have reported enhanced levels of muscle co-contraction when participants are learning new skills [11, 15, 45]. Enhanced levels of muscle co-contraction during gait are typically observed under challenging balance conditions [3, 22, 40] and in situations requiring enhanced joint stability [6]. In the present context, it may have served as a strategy to enhance leg stiffness and maximize balance during the treadmill condition. Alternatively, and as reported by Cappellini and colleagues for locomotion on a slippery surface, different muscle synergies may have emerged, reflecting the adoption of a new ‘gait mode’ as opposed to a mere adaptation for uncertain surface conditions [7]. Lastly, it should be noted that during treadmill walking, participants in the present study were constrained to the treadmill ring, to which they did not hold on, and wore the treadmill harness that did not provide any body weight support. Both the ring and harness, while inherent to the treadmill design, possibly provided haptic feedback to participants while walking. Such haptic feedback, in return, may have provided a stabilizing effect and reduced muscle activation amplitudes in the lower limbs [30]. If such effects of haptic feedback were present, however, it appears that they were not large enough to alleviate the larger levels of muscle activation amplitude observed during treadmill walking in the present study.

Omnidirectional treadmills are very promising since they allow not only changes in walking speed but also changes in walking trajectory. Coupled with VR technology, they can be used to train clinical populations on complex locomotor tasks as required for community ambulation. However, as the results suggest, there exist differences in gait while walking on the treadmill and overground. Further research is thus needed to see the impact of longer exposure on gait especially since gamers spend extended hours on it. The latter consideration is also important for rehabilitation to ensure an optimal transfer of training gains to situations of everyday life, and to avoid unwanted gait movements that would ultimately lead to pain and injury. Secondly, higher level of muscle activation observed in this study during treadmill walking could also result in higher energy consumption, which is consistent with the recent study done on other non-motorized low-cost omnidirectional treadmills [17].

### Limitations

A sample of convenience of young participants who have no sensorimotor impairments were included in the study. While this age range is not representative of the population at large, especially those typically seen in a rehabilitation setting, studying young participants gave benefit of understanding the influence of the omnidirectional treadmill on gait adaptations in the absence of other factors such as older age or a pathology affecting gait. This age group also represents the main users of omnidirectional treadmills which are primarily designed for entertainment purposes (video gaming). The performance of participants in the VE may also be shaped by the type of hardware and software used in this experiment (e.g. HMD, omnidirectional treadmill, etc.), and thus limit the generalization of the findings to another type of VR set up. Finally, while the present manuscript focused on speed adaptations, it is understood that one of the main advantages of the omnidirectional treadmill is the fact that it allows for direction changes. Participants of this study also took part in an experiment on locomotor steering and results will be presented in a different manuscript.

## Conclusion

The present study examined spatiotemporal parameters, body kinematics and lower limb muscle activation patterns while walking at different speeds on a non-motorized omnidirectional treadmill with and without VR vs. overground. Results show that participants achieved slower speeds and displayed differences in their walking pattern when ambulating on the omnidirectional treadmill compared to overground. Omnidirectional treadmill walking also yielded different walking adaptations in response to speed changes compared to overgound walking. Alterations of the walking pattern observed on the omnidirectional treadmill are reminiscent of those observed when walking on surfaces providing reduced shear forces and conditions that impose a threat to postural stability. Furthermore, the addition of VR to treadmill walking induced limited differences, suggesting that the treadmill itself is the main contributing factor to those alterations.

Non-motorized omnidirectional treadmills as the one examined in this study were primarily designed for entertainment purposes. Nevertheless, such treadmills show promise for rehabilitation and research inquiries given that they allow changing the speed and direction of walking in a safe and controlled environment and within a confined space. Given the walking alterations revealed in the present study, however, further research is needed to determine the impact of a prolonged use of the treadmill on gait in order to ensure an optimal transfer of training gains to situations of everyday life, and to avoid unwanted gait patterns that could ultimately lead to pain and injury.

## Data Availability

The datasets used and/or analysed during the current study are available from the corresponding author on reasonable request.

## Abbreviations

VR: Virtual reality
CRIR: Centre for interdisciplinary research in rehabilitation of greater Montreal
VEs: Virtual environments
EMG: Electromyography
RF: Rectus femoris
ST: Semitendinosus
TA: Tibialis anterior
MG: Medial gastrocnemius
PE: Physical environment
TV: Television
HMD: Head-mounted display
CoM: Center of mass
HF1: Hip flexion during early stance
HE: Peak hip extension
KF1: Maximal knee flexion during stance
KF2: Maximal knee flexion during swing
DF1: Peak ankle dorsiflexion at heel strike
DF2: Peak ankle dorsiflexion during mid stance
PF: Peak ankle planterflexion during late stance
GEE: Generalized estimating equation model
