# Correction to: Characterization of speed adaptation while walking on an omnidirectional treadmill

**DOI:** 10.1186/s12984-020-00796-x

**Published:** 2021-02-22

**Authors:** Smit Soni, Anouk Lamontagne

**Affiliations:** 1 Virtual Reality and Mobility Laboratory, Jewish Rehabilitation Hospital site of CRIR–CISSS de Laval, 3205 Place Alton-Goldbloom, Laval, H7V 1R2 QC Canada; 2 School of Physical and Occupational Therapy, McGill University, 3654 prom Sir-William-Osler, Montreal, H3G 1Y5 Canada

## Correction to: J NeuroEngineering Rehabil 10.1186/s12984-020-00787-y

The original article contains an error in Fig. 3 whereby the y-axis of the lower-left graph contains y-axis values that are mistakenly halved.

The corrected version of Fig. 3 can be viewed ahead with appropriately doubled y-axis values.

**Fig. 3:**
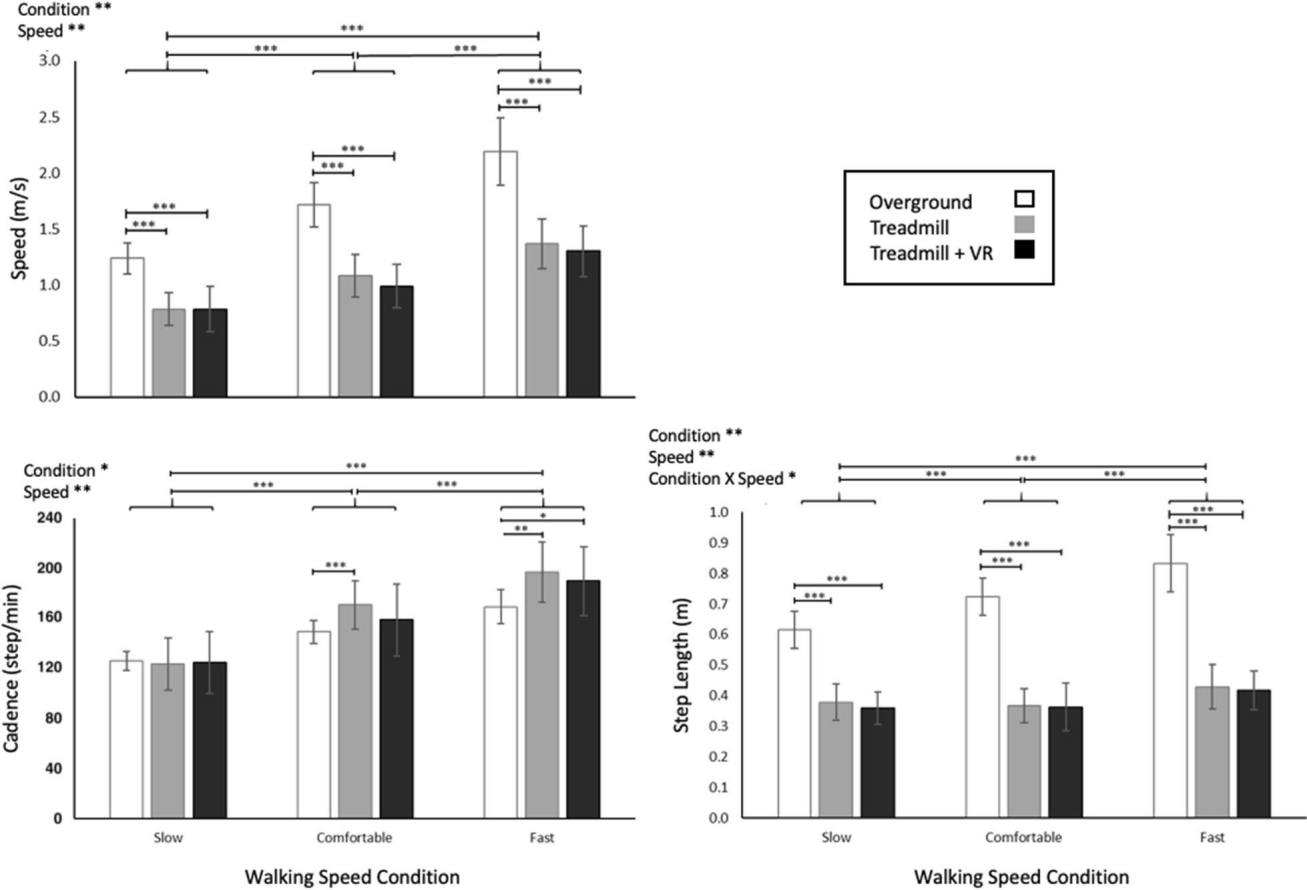
Average values (± 1SD) for gait speed, step length and cadence. Statistically significant main and interaction effects are indicated, as applicable. Likewise, post-hoc comparisons that were statistically significant are also illustrated. *p < 0.05. **p < 0.01. ***p < 0.0001

